# Assessment of respiratory dust exposure and lung functions among workers in textile mill (Thamine), Myanmar: a cross-sectional study

**DOI:** 10.1186/s12889-021-10712-0

**Published:** 2021-04-07

**Authors:** Thet Wai Oo, Mya Thandar, Ye Minn Htun, Pa Pa Soe, Thant Zaw Lwin, Kyaw Myo Tun, Zaw Myo Han

**Affiliations:** 1 Special Operation Medical Research Department, Defence Services Medical Research Centre, Nay Pyi Taw, Myanmar; 2 Department of Environmental and Occupational Health, University of Public Health, Yangon, Myanmar; 3 Department of Prevention and Research Development of Hepatitis, AIDS and Other Viral Diseases, Health and Disease Control Unit, Nay Pyi Taw, Myanmar; 4 Department of Preventive and Social Medicine, University of Medicine, Mandalay, Myanmar; 5 Department of Preventive and Social Medicine, Defence Services Medical Academy, Yangon, Myanmar; 6 Defence Services Liver Hospital, Yangon, Myanmar

**Keywords:** Dust exposure, Lung functions, Respiratory symptoms, Service duration, Textile worker

## Abstract

**Background:**

Airborne dusts are being potentially harmful for workers in occupational environment. Exposure to respirable dust is the most important concern in textile workers for the widespread of occupational lung diseases, especially more serious in developing countries. The aim of the study was to assess the respirable dust exposure and associated factors of lung functions among textile workers.

**Methods:**

A cross-sectional study was carried out at a textile mill (Thamine), Yangon Region, from April to December, 2018 and a total of 207 textile workers were randomly selected by using a multistage sampling procedure. Data were collected by using a structured questionnaire for respiratory symptoms, an air sampling pump for assessment of respirable dust exposure, and a spirometer for testing the lung functions. Logistic regression analysis was performed to assess the associated factors of lung functions. Odds ratios with a 95% confidence interval were computed for strength of associations at the significance level of α ≤ 0.05.

**Results:**

The mean (± standard deviation, SD) respirable dust exposure was 3.3 mg/m^3^ (± 0.69) and the prevalence of increased respirable dust exposure (> 3 mg/m^3^) was 50.7%. The level of respirable dust exposure was highest in the textile workers involving at twisting department. The means (± SD) spirometry values were FVC 82.8% (± 17.8), FEV_1_ 83.6% (± 18.5), and FEV_1_/FVC 0.9 (± 0.1). Overall magnitude of reduced lung functions was 40.1%, and the prevalence of reduced FVC, FEV_1,_ and FEV_1_/FVC were 36.7, 34.3 and 3.9% respectively. The current working at twisting department, > 5 years of service duration, respiratory symptoms and increased respirable dust exposure were associated with reduction in FVC and FEV_1_.

**Conclusions:**

The current working department, service duration, respiratory symptoms and exposure to respirable dust were predictors of lung functions in textile workers. An adequate ventilation, good work practices, hygienic workplace, safety and health training regarding potential health effects, and periodically assessment of lung functions are the critical elements for control of respirable dust exposure and reduction of occupational lung diseases.

**Supplementary Information:**

The online version contains supplementary material available at 10.1186/s12889-021-10712-0.

## Background

Occupational lung disease is a major concern and it has been listed as one of the priority problems in occupational health [1, 2]. In developing countries, workers are important as tools in production, and their health is at risk due to exposure to occupational hazards [2]. The textile industry is associated with a number of environmental problems such as water pollution, soil pollution, noise pollution, and air or dust pollution. Among these different textile pollutions, cotton dust pollution is the most important in terms of health effects on textile workers [3]. Dust are solid particles with a range in size from below 1 micron (μm) up to at least 100 μm. They may be or become airborne depending on their origin, physical characteristics, and ambient conditions [4].

The respirable dust is the fraction of the dust reaching the alveolar region of the lungs and it can penetrate beyond the terminal bronchioles to the gas exchange region of the lungs. Cotton dust is the dust present in the air during the handling or processing of cotton, which may contain a mixture of substances including ground-up plant matter, bacteria, fungi, soil, pesticides, and other contaminants [5]. Inhalation of the dust depends on its aerodynamic diameter, the velocity of the surrounding air, and the persons’ breathing rate. The small aerodynamic diameter has a greater chance of deep penetration into the respiratory tract, and dust with an aerodynamic diameter > 10 μm is easy to reach the gas-exchange region of the lung [4].

A large amount of dust was generated in different areas of the weaving section in the textile mill such as ginning, carding, and spinning operations [6]. The spinning section might be exposed to cotton dust more than the other sections. The textile workers were exposed to heavy dust concentration in the first step of processing [7]. The small dust particles entered into the alveoli of the lungs through inhalation. The capacity of retaining oxygen could be reduced by the accumulation of dust in the lymph causing damage to the alveoli [8]. The initial pulmonary responses to the dust might be characterized by reversible respiratory symptoms and deterioration in lung functions [9].

Exposure to occupational dust is implicated in the etiology of several occupational respiratory diseases with considerable socio-sanitary consequences [10]. The textile workers exposed to respiratory dust might cause a variety of different respiratory health problems including byssinosis, chronic obstructive pulmonary disease, and respiratory irritation [11, 12]. Cough, expectoration, and chest tightness were more prevalent in spinning and weaving workers [13]. The typical symptoms caused by exposure to respiratory dust were chronic cough with or without phlegm, dyspnea, wheezing, nasal stuffiness, and chest tightness [13–15]. The extent of chronic functional losses in textile workers was apparently affected by the consistency of reporting respiratory symptoms. It could be a stronger relationship between chronic respiratory diseases and spontaneous deteriorations in lung functions [15, 16].

In every industry, a safe workplace is crucial to achieving the highest productivity level. Therefore, the promotion and protection of a safe workplace is the complementary element of industrial development [17]. Finding and fixing workplace hazards before illness were the major concerns for safety and health at the workplace. Primary preventive interventions are important to reduce dust exposure in the workplace and remain vital for the elimination of the occupational lung disease burden [18]. Although the major improvements in dust control have occurred in many textile industries, a considerable proportion of textile workers are at risk of developing lung diseases, even at very low dust concentration in the workplace.

The studies conducted in Taiwan and Pakistan found that increased cotton dust concentration led to a reduction of lung functions in textile workers [8, 19]. In Myanmar, textile manufacturing is a labor-intensive industry and pivotal place in the economy. It represents one of the largest groups of manufacturing industries in terms of the value of production and source of direct employment to the people. However, the respiratory problems due to dust exposure is not well known and there is a limited scientific evidence for showing these kind of health issue in Myanmar. Therefore, the aim of the study was to assess respirable dust exposure and associated factors of lung functions among textile workers.

## Methods

### Study design and setting

A cross-sectional study was carried out among workers in a textile mill (Thamine), Yangon Region, Myanmar, between April and December 2018. There were 12 textile processing sections in this textile mill. The workers were working 8-h of work shift every day and wearing reusable cotton cloth face masks during working hours. As a ventilation condition, the working departments were equipped with general ventilation by installing the outlets at ceiling or exhaust fans, and local exhaust ventilation system.

### Study population

The study population was the textile workers who were working in six departments (weaving, knitting, opening, carding, spinning, and twisting) of the weaving section. Of these, the workers who did not give informed consent, and those who had heart diseases, tuberculosis, asthma and other respiratory diseases were excluded. The interview survey and lung function test were performed to all participants, and assessment of respiratory dust exposure was done at the personal breathing zones of the textile workers in each department. Smoking status was defined as a history of smoking occasionally or currently (cigarettes or cigars), or stopped smoking less than 6 months from the time of the study.

### Sampling technique and procedure

A single population proportion formula was used to calculate the sample size of the study. By using the prevalence of abnormal FEV_1_ (*p* = 0.16) from the study conducted in Greece [12], with an assumption of 95% confidence interval (CI), 5% margin of error, and 10% non-response rate, the final sample size was 207. A multistage sampling technique was used for this study. The weaving section was initially selected by purposive sampling. The total number of workers in six departments of the weaving section was 438. Secondly, the workers were selected using stratified random sampling, assuming that the workers in different departments would exhibit different level of exposure to respirable dust. Each department was taken as a stratum. The workers were allocated to each stratum proportionally to represent for their departments and selected by simple random sampling.

### Data collection procedures and data quality control

#### Interviews

A face-to-face interview was done by two interviewers using structured questionnaires. The questionnaire was developed by related literature (Additional file 1) and following an approved survey questionnaire of the Committee on Environmental and Occupational Health, British Medical Research Council [20]. The main contents of the questionnaire were background characteristics of participants (sex, age, educational status, current department, duration of service in current department, and smoking status) and the respiratory symptoms (cough, phlegm, cough with phlegm, rhinitis, wheezing, breathlessness, and chest illness). The initial version of the questionnaire was translated into the Burmese language and back-translated into English to verify accuracy. For the content validity, the questionnaire was pretested on 5% textile workers from Htet Aung garment factory that fulfilled the inclusion criteria. The inputs from the pretest were used to modify the questionnaire in more suitable contexts in order to generate intended data. The homogeneity of the questionnaire was fair to strong with high Cronbach α ranging from 0.70 to 0.83. The interview was conducted in a private setting and the consistency was checked before, during, and after entering the data.

#### Air sampling pump

An air sampling pump (AirChek 3000 Deluxe model 210–3311, SKC Limited, United Kingdom) was used for the assessment of respirable dust exposure in each department. It was performed by an occupational hygienist and placed at the personal breathing zones of the workers who were working with the cotton base materials. The two respirable dust samples were collected from each department. Samples were collected at a flow rate of 2.2 l/min during an 8-h work shift. All samples were collected on a Whatman glass microfiber filters having a 2.5 cm diameter to fit the sampling head. The weighting of the filter was on a calibrated analytical balance before and after sample collection, performed by a hygienist at the Occupational and Environmental Health Laboratory, Yangon.

#### Spirometry

A spirometer (Vitalograph In2itive model 2120, version 1.05, Vitalograph Limited, United Kingdom) was used for determining lung functions of the textile workers. Before testing, calibration of the spirometry was performed with the 3 L precision syringe. Firstly, Vitalograph compact flow head connected to the 3 L syringe and accuracy check monitor. The plunger was pulled out as far as possible before injecting the air and pumped the plunger into the syringe to inject the air. The results were automatically displayed and the system accuracy was checked again if the results were not within the acceptable range of 2.9–3.11 L.

After the accuracy check and explained the procedure to the participants, the lung function testing was performed by an occupational hygienist. The mouse piece was cleaned with antiseptic agents. In order to achieve the correct measurement of spirometry, the occupational hygienist made sure that the posturing of the participants was done by sitting upright, touching feet flat on the floor with legs uncrossed, losing tight-fitting clothing, leaving dentures in the mouth normally, and using a chair with arms. Before performing the procedure, the participants had instructed to practice deep inspiration and complete forceful expiration. The highest values for forced vital capacity (FVC) and forced expiratory volume in the first second (FEV_1_) after three acceptable maneuvers were used in subsequent analysis.

### Measurement of variables

A participant presenting at least one symptom was considered as the symptomatic person in this study. The duration of service in the current department was the total number of working years in the current department of the weaving section. If a decimal was greater than 6 (months), the length was counted as 1 (year) and equal 0 if less than 6 (months). The concentration of respirable dust exposure was classified at > 3 mg/m^3^ of 8 h mean dust concentration as increase exposure, and ≤ 3 mg/m^3^ as acceptable exposure [21]. The concentration of respirable dust exposed to workers was calculated according to the following equation:

$$ \mathrm{Concentration}\ \left(\mathrm{mg}/\mathrm{m}3\right)=\frac{\mathrm{Final}\ \mathrm{weight}\ \left(\mathrm{mg}\right)-\mathrm{Initial}\ \mathrm{weight}\ \left(\mathrm{mg}\right)}{\mathrm{Time}\ \left(\min \right)\times \mathrm{flow}\ \mathrm{rate}\ \left(\mathrm{litre}/\min \right)}\times \mathrm{1,000} $$

The lung functions of the participants were measured by a hygienist and expressed as percent prediction. FVC was defined as the total expiratory volume from one time of a maximally forced expiration maneuver. FEV_1_ was defined as expiratory volume that has been exhaled at the end of the first second of a maximally forced expiration maneuver. FEV_1_/FVC was defined as the ratio of FEV_1_ and FVC which was expressed by the American Thoracic Society/Europe Respiratory Society Best (ATS/ERS Best), and it provided the identification of obstructive or restrictive impairment. The normal lung function was defined as ≥80% of predicted FVC, ≥ 80% of predicted FEV_1_, and ≥ 0.7 of FEV_1_/FVC. The abnormal lung function was categorized as restrictive and obstructive impairments. Restrictive impairment was characterized by a normal FEV_1_/FVC with reduced FVC and normal or reduction in FEV_1_. The obstructive impairment was characterized by a reduction in FEV_1_/FVC with reduced or normal FVC, and reduced FEV_1_ [22, 23].

### Data processing and analysis

The data were coded, entered into a Microsoft Excel sheet (version 2016), and checked for errors. Data analysis was performed by using the International Business Machines Corporation-Statistical Package for Social Sciences program version 23. The mean and standard deviation (SD) were expressed for the result of a continuous variable, whereas frequency and percentage were used to display the result of the categorical variable. The normality of the continuous data was viewed by using the Kolmogorov–Smirnov test and the data distribution was normal. The differences between means of respirable dust concentration in six departments were calculated by using one-way analysis of variance with a post-Hoc Tukey HSD test. Bivariate logistic regression analysis was performed to identify the associated factors of dependent variable, reduced lung functions. The variables with significant association were identified on the basis of odd ratio (OR) with 95% confidence interval (CI) and *p* value ≤0.05.

## Results

### Background characteristics of textile workers

A total of 207 workers from six departments were selected in this study. As shown in Tables 1, 90.8% were women and only 9.2% were men. The mean (± SD) age was 38.8 (± 11.2) years with a range of 18–60 years and 29.5% of textile workers were age group of ≤30 years. For educational level, 44.9% attained a high school education, and 9.7% were graduate and above. Among the total, 37.7% were in the weaving department, each of 19.3% were involving in the spinning and twisting departments, respectively, and 5.8% were in opening department. The mean duration of service in the current department was 13.4 (SD ± 10.6) years with a range of 0.5–39.0 years and 40.1% had more than 15 years of service duration in the current department. Of all workers, 3.9% were smokers and 96.1% had no history of smoking.

**Table 1:** Background characteristics of textile workers

Variables	Frequency (%)
Sex	
Male	19 (9.2)
Female	188 (90.8)
Age (year)	
≤ 30	61 (29.5)
31–40	52 (25.1)
41–50	56 (27.1)
> 50	38 (18.4)
Mean ± SD (38.8 ± 11.2) years, Minimum 18 years, Maximum 60 years	
Educational status	
Read and write	3 (1.4)
Primary school education	18 (8.7)
Middle school education	62 (30.0)
High school education	93 (44.9)
College or University	11 (5.3)
Graduate and above	20 (9.7)
Current department	
Weaving	78 (37.7)
Knitting	24 (11.6)
Carding	13 (6.3)
Spinning	40 (19.3)
Twisting	40 (19.3)
Opening	12 (5.8)
Duration of service in current department (year)	
≤ 5	54 (26.1)
6–10	54 (26.1)
11–15	16 (7.7)
> 15	83 (40.1)
Mean ± SD (13.4 ± 10.6) years, Minimum 0.5 year, Maximum 39.0 years	
Smoking status	
No	199 (96.1)
Yes	8 (3.9)

### Prevalence of respiratory symptoms among textile workers

Table 2 showed the respiratory symptoms of textile workers. Among the total participants, 52.7% had respiratory symptoms. As for specific respiratory symptoms, 8.7% reported cough, 21.3% phlegm, 9.2% cough and phlegm, 15.9% rhinitis, 10.1% wheezing, 34.3% breathlessness and 4.8% chest illness.

**Table 2:** Prevalence of respiratory symptoms among textile workers

Respiratory symptoms	Frequency (%)
Respiratory symptoms	
Absent	98 (47.3)
Present	109 (52.7)
Cough	
Absent	189 (91.3)
Present	18 (8.7)
Phlegm	
Absent	163 (78.7)
Present	44 (21.3)
Cough with phlegm	
Absent	188 (90.8)
Present	19 (9.2)
Rhinitis	
Absent	174 (84.1)
Present	33 (15.9)
Wheezing	
Absent	186 (89.9)
Present	21 (10.1)
Breathlessness	
Absent	136 (65.7)
Present	71 (34.3)
Chest illness	
Absent	197 (95.2)
Present	10 (4.8)

### Respirable dust exposure and lung functions among textile workers

**Fig. 1:**
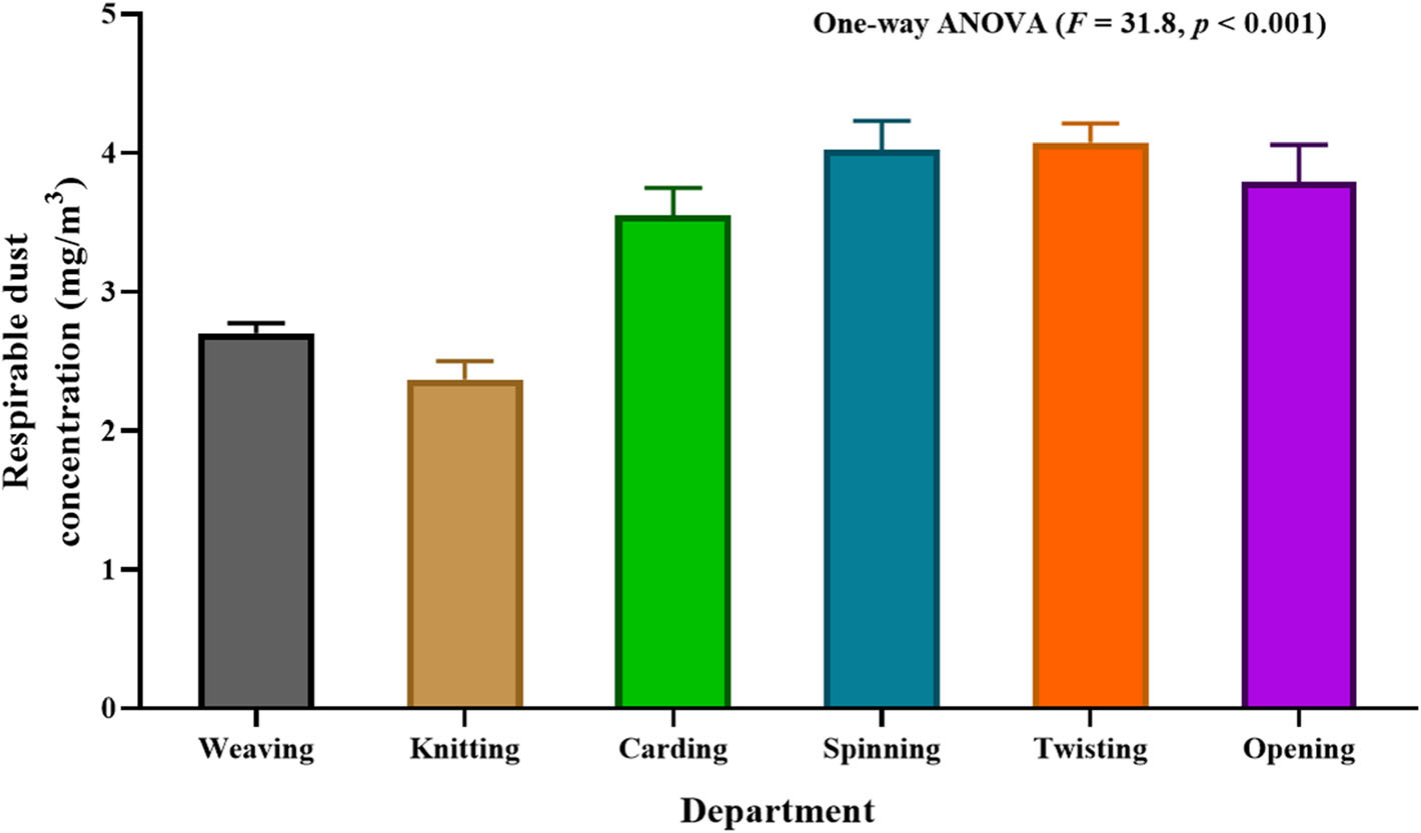
Respirable dust concentration of textile workers in each department of weaving section

As shown in Table 3, 50.7% of textile workers exposed to > 3 mg/m^3^ of respirable dust exposure. The mean (± SD) respirable dust exposure was 3.3 (± 0.7) mg/m^3^ with a range of 2.4–4.1 mg/m^3^. The respirable dust concentration in each department was shown in Fig. 1. The means (± SD) respirable dust concentration were 2.7 (± 0.1) mg/m^3^ in the weaving department, 2.4 (± 0.1) mg/m^3^ in the knitting department, 3.6 (± 0.2) mg/m^3^ in the carding department, 4.0 (± 0.2) mg/m^3^ in the spinning department, 4.1 (± 0.1) mg/m^3^ in the twisting department and 3.8 (± 0.3) mg/m^3^ in the opening department. The difference of means respirable dust exposure among six departments was statistically significant, *F* (5, 6) = 31.8 and *p* <  0.001.

**Table 3:** Respirable dust exposure and lung functions among textile workers

Variables	Frequency (%)
Respirable dust exposure	
Acceptable (≤ 3 mg/m^3^)	102 (49.3)
Increased (> 3 mg/m^3^)	105 (50.7)
Mean ± SD (3.3 ± 0.7), Minimum 2.4, Maximum 4.1	
Lung functions	
Normal	124 (59.9)
Reduced	83 (40.1)
Restrictive	75 (90.4)
Obstructive	8 (9.6)
FVC (% prediction)	
Normal (≥ 80%)	131 (63.3)
Reduced (< 80%)	76 (36.7)
Mean ± SD (82.8 ± 17.8), Minimum 26, Maximum 134	
FEV_1_ (% prediction)	
Normal (≥ 80%)	136 (65.7)
Reduced (< 80%)	71 (34.3)
Mean ± SD (83.6 ± 18.5), Minimum 22, Maximum 149	
FEV_1_/FVC	
Normal (≥ 0.7)	199 (96.1)
Reduced (< 0.7)	9 (3.9)
Mean ± SD (0.9 ± 0.1), Minimum 0.6, Maximum 1.0

Concerning lung functions of textile workers, 40.1% had reduced lung functions, of which 90.4% had restrictive impairment and 9.6% had obstructive impairment. The mean (± SD) of FVC was 82.8% (± 17.8) with a range of 26–134 and 36.7% of participants presented a reduction in FVC. The mean (± SD) of FEV_1_ was 83.6% (± 18.5) with a range of 22–149 and 34.3% of participants presented a reduction in FEV_1_. For the FEV_1_/FVC, the mean (± SD) was 0.9 (± 0.1) with a range of 0.6–1.0 and 3.9% of participants presented a reduction in it.

### Factors associated with lung functions among textile workers

As shown in Table 4, the workers involved in twisting department (OR = 3.00, 95% CI = 1.35–6.66), those who had 6–10 years of service duration (OR = 4.60, 95% CI = 1.83–11.58), those who had 11–15 years of service duration (OR = 7.39, 95% CI = 2.14–25.56), those who had > 15 years of service duration (OR = 4.19, 95% CI = 1.76–9.98), those who were with respiratory symptoms (OR = 4.12, 95% CI = 2.22–7.64), those who had increased exposure of respirable dust (OR = 2.04, 95% CI = 1.14–3.63) were significantly associated with reduction in FVC. Likewise, the workers involved in twisting department (OR = 3.43, 95% CI = 1.53–7.69), those who had 6–10 years of service duration (OR = 2.80, 95% CI = 1.16–6.73), those who had 11–15 years of service duration (OR = 3.42, 95% CI = 1.03–11.39), those who had > 15 years of service duration (OR = 2.90, 95% CI = 1.29–6.56), those who were with respiratory symptoms (OR = 2.82, 95% CI = 1.54–5.17), those who had increased exposure of respirable dust (OR = 1.83, 95% CI = 1.02–3.29) were significantly associated with reduction in FEV_1_.

**Table 4:** Factors associated with lung functions among textile workers

Variables	Total	FVC		FEV_1_		FEV_1_/FVC	
		Reduced n (%)	OR (95% CI)	Reduced n (%)	OR (95% CI)	Reduced n (%)	OR (95% CI)
Gender							
Male	19	6 (31.6)	1.00	6 (31.6)	1.00	2 (10.5)	1.00
Female	188	70 (37.2)	1.29 (0.47–3.53)	65 (34.6)	1.15 (0.42–3.15)	6 (3.2)	0.28 (0.05–1.48)
Age							
< 40 years	109	35 (32.1)	1.00	34 (31.2)	1.00	3 (2.8)	1.00
≥ 40 years	98	41 (41.8)	1.52 (0.86–2.68)	37 (37.8)	1.34 (0.75–2.38)	5 (5.1)	1.90 (0.44–8.17)
Educational level ^a^							
≥ High school education level	124	41 (33.1)	1.00	39 (31.5)	1.00	4 (3.2)	1.00
< High school education level	83	35 (42.2)	1.48 (0.83–2.62)	32 (38.6)	1.36 (0.76–2.45)	4 (4.8)	1.52 (0.36–6.25)
Current department							
Weaving	78	21 (26.9)	1.00	19 (24.4)	1.00	3 (3.8)	1.00
Knitting	24	8 (33.3)	1.36 (0.51–3.64)	9 (37.5)	1.86 (0.70–4.94)	1 (4.2)	1.09 (0.11–10.96)
Carding	13	4 (30.8)	1.21 (0.34–4.34)	4 (30.8)	1.38 (0.38–4.99)	0 (0.0)	0.00
Spinning	40	16 (40.0)	1.81 (0.81–4.05)	13 (32.5)	1.49 (0.65–3.46)	2 (5.0)	1.32 (0.21–8.21)
Twisting	40	21 (52.5)	3.00 (1.35–6.66) **	21 (52.5)	3.43 (1.53–7.69) **	1 (2.5)	0.64 (0.06–6.37)
Opening	12	6 (50.0)	2.71 (0.79–9.35)	5 (41.7)	2.22 (0.63–7.81)	1 (8.3)	2.27 (0.22–23.83)
Service duration							
≤ 5 years	54	8 (14.8)	1.00	10 (18.5)	1.00	0 (0.0)	
6–10 years	54	24 (44.4)	4.60 (1.83–11.58) ***	21 (38.9)	2.80 (1.16–6.73) *	3 (5.6)	
11–15 years	16	9 (56.3)	7.39 (2.14–25.56) **	7 (43.8)	3.42 (1.03–11.39) *	1 (6.3)	
> 15 years	83	35 (42.2)	4.19 (1.76–9.98) ***	33 (39.8)	2.90 (1.29–6.56) **	4 (4.8)	
Smoking status							
No	199	74 (37.2)	1.00	70 (35.2)	1.00	8 (4.0)	
Yes	8	2 (25.0)	0.56 (0.11–2.86)	1 (12.5)	0.26 (0.03–2.18)	0 (0.0)	
Respiratory symptoms							
Absent	98	20 (20.4)	1.00	22 (22.4)	1.00	5 (5.1)	1.00
Present	109	56 (51.4)	4.12 (2.22–7.64) ***	49 (45.0)	2.82 (1.54–5.17) ***	3 (2.8)	0.53 (0.12–2.26)
Respirable dust exposure							
Acceptable	102	29 (28.4)	1.00	28 (27.5)	1.00	4 (3.9)	1.00
Increased	105	47 (44.8)	2.04 (1.14–3.63) *	43 (41.0)	1.83 (1.02–3.29) *	4 (3.8)	0.97 (0.24–3.99)

^a^ Education level - categorized as ≥ high school education level (high school education, college/university, and graduate and above) and < high school education level (read and write, primary school education, and middle school education)

* *p* < 0.05, **p < 0.01, *** *p* < 0.001

## Discussion

This study has assessed the respirable dust exposure and associated factors of lung functions in textile workers. Almost all textile workers were female, and the male-female ratio was 1:10 in this study. It was similar to the result of the study carried out in Thailand stated that the majority of participants were female [24], while the study conducted in Ethiopia reported that there was no difference in sex distribution [25]. It might be due to different study areas and the nature of employment which means that the textile industry is generally or traditionally viewed as suitable employment for female workers in Myanmar. The mean age of textile workers was 38.8 years and it was consistent with the studies done in Thailand [24] and Nigeria [26] reported that the means age of participants were 39.7 and 36.9 years, respectively. In this study, most of the textile workers had a high school education level, but other studies conducted in the same constitution of India [27] and Ethiopia [28] stated that most of the participants were up to high school and illiterate, respectively.

For the duration of service in the current department, most of the textile workers had more than 15 years of service duration. In contrast, a study conducted in India reported that most participants had more than 20 years of service duration [27] and, in an Ethiopia study, most of the participants were exposed to the dust in the current section for 20 to 30 years [25]. In reported respiratory symptoms, 52.7% of textile workers had at least one respiratory symptom, and it was lower than the finding of a study done in Egypt showed that 59% of participants had respiratory symptoms. However, it was higher than the findings of the same studies conducted in Taiwan [8] and Ethiopia [29] reported that and 39.7 and 47.8% of participants had respiratory symptoms respectively.

The occupational lung diseases were rapid outsourcing of textile industries due to the long exposure period in the workplace and poor control measures. The textile workers who were exposed to dust reported respiratory symptoms as a result of hypersensitive airways and an acute reduction in FEV_1_ [30]. Most of the acute respiratory symptoms due to dust exposure in textile workers were chest tightness, cough, and dyspnea [29]. In this study, breathlessness 34.3%, and phlegm 21.3% were the commonest respiratory symptoms reported by the textile workers. The reported respiratory symptoms of textile workers in the same occupational setting varied substantially from different studies. In contrast with other findings, a study conducted in Thailand revealed that there was a high prevalence of chest tightness 64.6% and phlegm 59.1% among the participants [10]. A Pakistan study stated that the wheezing 20% and phlegm 20% were the most reported respiratory symptoms of textile workers [19].

In this study, the mean respirable dust concentration was 3.30 mg/m^3^, and 50.7% of textile workers were exposed to an increased level of respirable dust concentration. This finding was higher than the study done in Thailand showed that the mean respirable dust concentration was 0.53 mg/m^3^ in sewing workers [10]. It might be due to the fact that the textile workers in the weaving section exceeded occupational dust exposure. In this study, the mean respirable dust concentration in the twisting department was more than the other departments of the weaving section. In contrast, the carding department was an area of highest dust concentration in the studies done in Ethiopia [25, 31] and Taiwan [8]. Additionally, a similar study reported that the dust concentration was varied between the working departments and the level of dust concentration was higher in the cleaning department than in the spinning and weaving departments [28]. This inconsistency in the results might be, in part, due to the methodological variation, the difference in an industrial setting, and the handling and processing of cotton.

In this study, the prevalence of reduced lung functions, 40.1%, among textile workers was higher compared with the other study done in Taiwan reported that 38.5% had reduced lung functions. Regarding spirometry patterns, 36.2% of textile workers had the restrictive pattern and it was lower than a similar study done in Nigeria found that the prevalence of restrictive pattern was 40.0% among textile workers [26]. There were 3.9% of an obstructive pattern among textile workers in this study, but conversely, the studies conducted in Zimbabwe [32] and Nigeria [26] reported that 27.8 and 10.0% of study participants had the obstructive pattern. The mean value of FVC, 82.82%, was lower than the studies done in the same occupational setting at Thailand 106.0%, Greece 90.6%, and Pakistan 90.3% [10, 12, 19]. The mean value of FEV_1_, 83.64%, was coincided with the result of a study conducted in Pakistan [19], however, it was lower than the studies done in Thailand 101.0%, Iran 88.8%, Turkey 96.2%, and Greece 91.7% [10, 12, 33, 34]. This inconsistency of results might be attributable to the usage of different types of spirometers in these studies.

As a current working department, respirable dust concentration was highest in the twisting department, and the textile workers who were working in this department were the more likely to reduce lung functions compared with those who were working in other departments. The various conditions such as the quality of the cotton, the production rate, the ventilation system, the processing method, and the method of dust sampling and analysis might affect the concentration of dust in the working environment [31]. The direct exposure to dust might contribute to reduced lung functions, and pulmonary diseases due to occupational exposure are mostly related to inhalation of dust and its deposition in the lungs [13].

The duration of service in the current department was associated with lung functions and the textile workers who were exposed to dust > 5 years were more likely to reduce their lung functions than those who had ≤ 5 years of service duration. This finding was also in line with the study conducted in India reported that cotton mill workers with > 5 years of dust exposure were more likely to have spirometric abnormality [29]. Consequently, the textile workers with long service duration and chronic exposure to dust were at high risk of developing chronic respiratory health problems [35]. However, the studies conducted in the same constitutions of Egypt and Nigeria showed that there was no correlation between spirometric functions and duration of exposure to dust in the current section among cotton textile workers [13, 26]. It might be due to the variation of the working environment and concentration of dust exposure in workplaces.

The textile workers with respiratory symptoms had 3.64 times more likely to reduce respiratory function than those who had no respiratory symptoms. The result of the current study was in agreement with a study done in Nigeria showed that the textile workers with respiratory symptom had low FEV_1_ predicted value and a possibility of obstructive airway disease [26]. This finding was also matched with the study done in India stated that spirometric abnormality was more prevalent in symptomatic workers and there was an association between spirometry results and respiratory symptoms in cotton mill workers [29]. This might be due to increased dust exposure in the working environment and the accumulation of dust in the respiratory system. Dust particles or dust-containing macrophages causing injury to the lungs, and then fibrous lung tissue provided functional impairment.

The spirometric data of the current study showed a significant reduction of lung functions that occurred in most textile workers who were exposed to increased respirable dust concentration. It was consistent with the finding of the Taiwan study reported that more prevalence of impaired lung functions occurred in the cotton textile workers with higher exposure to dust [8]. This finding also supported to the results of the study done in Pakistan showed that mean dust exposure level affects on lung functions of textile workers, and increased dust concentration led to more decline in lung functions of textile workers [30]. This consistent finding provided strong evidence of increased respirable dust exposure that was an associated factor of reduced lung functions.

The smoking habit was not associated with lung functions in this study resulting from a low distribution of workers with smoking history. The results of this study might be generalized to elsewhere in which the workers are involved in the same occupational setting. However, the results might be varied depending on the diversity of basic characteristics, risk behaviors, respirable dust exposure, and implementation of occupational safety measures. There were some limitations to this study. The first was related to healthy worker effect which means that the workers who developed the respiratory symptoms may have quitted the job. Airborne endotoxin is more responsible for occupational respiratory diseases than respiratory dust itself and so, lack of the measurement of endotoxin exposure in the workplace was the second limitation of this study. Thirdly, the cross-sectional study could not determine the cause and effect relationship and therefore it would require further prospective studies in order to assess the causality and confirm the findings of this study. Finally, the interview by using the questionnaire method was another limitation because it may cause recall bias and interviewer bias. Despite all of these limitations, this study might be a reasonable source of information for occupational health and safety.

## Conclusions

The respirable dust concentration was the highest in the twisting department of the weaving section. The textile workers involved in the twisting department, those who were more than 5 years of service duration, and those who had increased respirable dust exposure were at risk for reduced lung functions. A total enclosure of the early stages of cotton processing and ventilation system that keeps the enclosure under negative pressure is needed to reduce respirable dust exposure. The industry administration should arrange the safety and health training regarding potential health effects of respirable dust exposure, preventive measures in the workplace, good work practices, performance assessment and workplace environment measures. The employers should explore the engineering control measure, occupational safety policies, and administrative controls to reduce respirable dust exposure. Additionally, periodically assessment of lung functions with spirometry should be applied to textile workers for screening the occupational lung diseases. The government should provide the funding or technical support to textile industries for the installation of new technologies and make the law and policies for checking the level of dust concentration daily or weekly in basis.

## Supplementary Information

**Additional file 1.** English language version of the questionnaire.

## Data Availability

The data analyzed for this manuscript are available from the corresponding author and can be made accessible upon reasonable request.

## Abbreviations

CI: Confident Interval
FEV_1_: Forced Expiratory Volume in the First Second
FVC: Forced Vital Capacity
OR: Odd Ratio
SD: Standard Deviation
