# Supplementary material for: Assessment of respiratory dust exposure and lung functions among workers in textile mill (Thamine), Myanmar: a cross-sectional study
Source: BMC Public Health. 2021 Apr 7;21:673. doi: 10.1186/s12889-021-10712-0 (PMC8028193; doi:10.1186/s12889-021-10712-0)
Supplement: Supplementary file 1 — Additional file 1. English language version of the questionnaire. [file 12889_2021_10712_MOESM1_ESM.docx]

**QUESTIONNAIRES**

**Code**

| **Sociodemographic characteristics** | | | | | | | | | | | | | |
| --- | --- | --- | --- | --- | --- | --- | --- | --- | --- | --- | --- | --- | --- |
| Age (completed) ……………… | | | | years | Sex | | | | 🞎 Male | | | | 🞎 female |
| Education | |  |  | | | | |  | | |  | | |
|  | 🞎 Illiterate | | | | | 🞎 High school education level | | | | | | | |
|  | 🞎 Read and write | | | | | 🞎 College or university | | | | | | | |
|  | 🞎 Primary school education level | | | | | 🞎 Graduate and above | | | | | | | |
|  | 🞎 Middle school education level | | | | |  | | | | | | | |
| Current department | | | | | | | | | | | | | |
|  | 🞎 Weaving | | | | | | 🞎 Spinning | | | | | | |
|  | 🞎 Knitting | | | | | | 🞎 Twisting | | | | | | |
|  | 🞎 Carding | | | | | | 🞎 Opening | | | | | | |
| Duration of service in current department …………………… years | | | | | | | | | | | | | |
| Smoking status | | | | | | | | | | 🞎 Yes | | 🞎 No | |
| **Respiratory symptoms** | | | | | | | | | | | | | |
| Do you experience respiratory symptoms? | | | | | | | | | | 🞎 Present | | 🞎 Absent | |
| *If present, please answer the following question(s).* | | | | | | | | | | | | | |
| **Cough** | | | | | | | | | | | | | |
| Do you cough early morning or during the day or at night on most days for as much as three months each year? | | | | | | | | | | 🞎 Present | | 🞎 Absent | |
| **Phlegm** | | | | | | | | | | | | | |
| Do you bring up phlegm early morning or during the day or at night on most days for as much as three months each year? | | | | | | | | | | 🞎 Present | | 🞎 Absent | |
| **Cough with phlegm** | | | | | | | | | | | | | |
| Do you cough with phlegm early morning or during the day or at night on most days for as much as three months each year? | | | | | | | | | | 🞎 Present | | 🞎 Absent | |
| **Rhinitis** | | | | | | | | | | | | | |
| Have you had sneezing and stuffiness on most days for as much as three months each year? | | | | | | | | | | 🞎 Present | | 🞎 Absent | |
| **Wheezing** | | | | | | | | | | | | | |
| In the past one year, have you had attacks of wheezing or whistling from your chest? | | | | | | | | | | 🞎 Present | | 🞎 Absent | |
| **Breathlessness** | | | | | | | | | | | | | |
| Are you troubled by shortness of breath when hurrying on level ground or walking at a slight hill? | | | | | | | | | | 🞎 Present | | 🞎 Absent | |
| **Chest illness** | | | | | | | | | | | | | |
| In the past one year, have you had any chest illness which has kept you away from your work for as much as a week? | | | | | | | | | | 🞎 Present | | 🞎 Absent | |
